# Mechanisms of three-dimensional growth of thyroid cells during long-term simulated microgravity

**DOI:** 10.1038/srep16691

**Published:** 2015-11-18

**Authors:** Sascha Kopp, Elisabeth Warnke, Markus Wehland, Ganna Aleshcheva, Nils E. Magnusson, Ruth Hemmersbach, Thomas Juhl Corydon, Johann Bauer, Manfred Infanger, Daniela Grimm

**Affiliations:** 1 Clinic for Plastic, Aesthetic and Hand Surgery, Otto-von Guericke-University, 39120 Magdeburg, Germany; 2 DLR German Aerospace Centre, Department of Gravitational Biology, 51147 Cologne, Köln, Germany; 3 Department of Biomedicine, Aarhus University, DK-8000 Aarhus C, Denmark; 4 Medical Research Laboratory, Department of Clinical Medicine, Aarhus University, 8000 Aarhus C, Denmark; 5 Max-Planck-Institute of Biochemistry Martinsried, 82152 Martinsried, Germany

## Abstract

Three-dimensional multicellular spheroids (MCS) of human cells are important in cancer research. We investigated possible mechanisms of MCS formation of thyroid cells. Both, normal Nthy-ori 3–1 thyroid cells and the poorly differentiated follicular thyroid cancer cells FTC-133 formed MCS within 7 and 14 days of culturing on a Random Positioning Machine (RPM), while a part of the cells continued to grow adherently in each culture. The FTC-133 cancer cells formed larger and numerous MCS than the normal cells. In order to explain the different behaviour, we analyzed the gene expression of *IL6, IL7, IL8, IL17, OPN, NGAL*, *VEGFA* and enzymes associated cytoskeletal or membrane proteins (*ACTB*, *TUBB*, *PFN1, CPNE1, TGM2, CD44, FLT1*, *FLK1, PKB, PKC, ERK1/2, Casp9, Col1A1)* as well as the amount of secreted proteins (IL-6, IL-7, IL-8, IL-17, OPN, NGAL, VEGFA). Several of these components changed during RPM-exposure in each cell line. Striking differences between normal and malignant cells were observed in regards to the expression of genes of *NGAL*, *VEGFA*, *OPN*, *IL6* and *IL17* and to the secretion of VEGFA, IL-17, and IL-6. These results suggest several gravi-sensitive growth or angiogenesis factors being involved in 3D formation of thyroid cells cultured under simulated microgravity.

Space travellers suffer of a variety of health problems^1^,^2^,^3^, such as bone loss, muscle atrophy, changes in metabolism, cardiac problems and others. In addition, reduced thyroid hormone levels were found in the plasma of rats^4^ and astronauts returning from a space mission^5^ as well as in the supernatants of thyroid cancer cells cultured on a Random Positioning Machine (RPM)^6^. Besides the direct impact of microgravity (μ*g*) on the human body, an influence on the cellular level has been found under altered gravity conditions. *In vitro*, simulated microgravity (s-μ*g*) induces three-dimensional (3D) growth in many cell types^3^,^7^. Even though monolayer culture models are easy and convenient to set up with good viability of cells in culture, they lack the 3D microenvironment^8^. The 3D culture of various cell types reflects the *in vivo* situation more precisely than two-dimensional (2D) cell culture techniques. Moreover, spheroids produce their own extracellular matrix (ECM) over time that differs in the relative amount and assembly from the corresponding monolayer cultures. Many cell types in 3D multicellular culture models were found to assume a more or less normal cellular architecture and exhibited gene expression profiles that were reflective of an authentic differentiated phenotype found in the real tissue^3^,^8^. By means of this experimental approach, mechanisms and pathways can be studied that control cancer cell growth and function^9^. 3D aggregates of cancer cells represent a simple model of a tumour. These multicellular spheroids (MCS) mimic small metastases and areas of solid tumours *in vivo*^10^. Changes occurring during 3D spheroid formation are not known in detail. Up to now cytoskeletal reorganizations^11^,^12^,^13^,^14^,^15^,^16^, altered gene expression and cytokine secretion^10^,^17^,^18^ and apoptosis^10^,^14^,^19^,^20^ have been described in the literature. But it is still unknown how the cells are able to sense altered gravity conditions, although current research hints in the direction of cytoskeletal changes.

Therefore, further experiments on cells cultured under microgravity are required. For this purpose devices simulating microgravity on Earth are mainly used, because possibilities of performing experiments in Space are rare. In this study we used the RPM, which prevents sedimentation by randomization of the *g*-vector^21^,^22^ and exposed normal and poorly differentiated thyroid carcinomas (PDTC). PDTC is a distinct diagnostic entity and its incidence is 2–3% of thyroid cancers in North America^23^.

There are several cell lines, which enable meaningful research on this disease. The FTC-133 cell line was derived from a PDTC from a 42-year-old male patient^24^. It comprises follicular thyroid cancer cells, which formed large 3D aggregates, when cultured under real microgravity (r-μ*g*) and simulated μ*g*^10^,^18^,^25^. In this paper we investigated FTC-133 cultured for 7 and 14 days on the RPM in comparison to non-malignant human thyroid cells of the line Nthy-ori 3–1. It is a primary thyroid follicular epithelial cell line originating from human tissue, which was stably transfected with plasmid DNA containing an origin-defective simian-virus 40 (SV-40) genome^26^. The immortalized cell line inherits thyroid specific functions, namely iodide-trapping and thyroglobulin production, and showed no tumorigenesis when transplanted into nude mice^26^.

The principal aim of this study was firstly to investigate the changes of poorly differentiated follicular thyroid cancer cells and normal thyroid cells, when they are cultured under simulated microgravity conditions. A similar reaction of both cell types to the microgravity environment may be due to a general reaction of cells to low-gravity conditions.

In addition, we examined the differences in the formation of MCS of normal cells versus cancer cells on the RPM. We focused on specific cytokines, adhesion molecules, growth factors, cytoskeletal proteins or proteins which are necessary for growth and metastasis.

Healthy Nthy-ori 3–1 cells and FTC-133 follicular thyroid cancer cells were exposed to simulated microgravity on the RPM for different time periods in order to investigate differences in the formation of multicellular spheroids. We expected that finding similarities might hint to triggers of MCS formation, whereas differences will increase our knowledge in cancer biology. In addition, we characterized 3D multicellular spheroids tissue-engineered on the RPM and examined the cytokine secretion by the thyroid cells. The study revealed that several factors promoting angiogenesis such as interleukin-6 (IL-6), vascular endothelial growth factor (VEGFA), and neutrophil gelatinase-associated lipocalin (NGAL) are sensitive to simulated microgravity in thyroid cells cultured on the RPM.

## Results

### Multicellular spheroid formation during RPM-exposure

**Figure 1:**
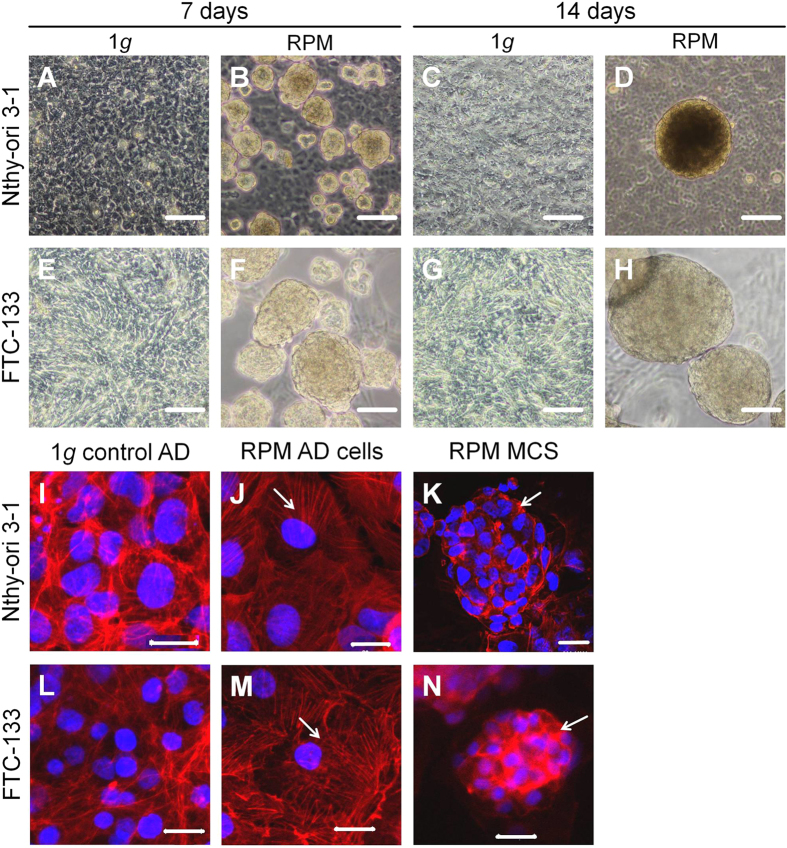
Morphology of the cells cultured on the RPM. Phase-contrast microscopy of Nthy-ori 3-1 (**A**–**D**) and FTC-133 (**E**–**H**) after culturing on the RPM for 7 and 14 days and corresponding 1*g* control cells: Control samples from Nthy-ori 3-1 (**A**,**C**) as well as from FTC-133 (**E**,**G**) showed no formation of multicellular spheroids. Culturing of the cells on the RPM, however, triggered a moderate formation of MCS for Nthy-ori 3-1 cells (**B**,**D**) and a massive aggregate formation of FTC-133 (**F**,**H**). Scale bar: 100 μm. Confocal laser scanning microscopy of Nthy-ori 3-1 (**I**–**K**) and FTC-133 (**L**–**N**) after a 7-day-exposure on the RPM and their corresponding 1*g* control cells: The pictures show control cells (**I**,**L**), AD cells (**J**,**M**) and multicellular spheroids (**K**,**N**) after a 7-day-exposure. Scale bar: 20 μm; blue staining: DAPI highlights the nucleus; red staining: phalloidin Texas red to visualize the F-actin. Arrows indicate region of interest (actin cytoskeleton).

Observations of the morphology of both cell lines were performed by phase contrast microscopy after a 7- and 14-day-exposure to simulated microgravity (s-μ*g*) on the RPM (Fig. 1A–H).

#### Nthy-ori 3–1

Cells of the non-rotated control samples (static controls) grew adherently (1*g*) in 2D monolayer cultures and formed no 3D aggregates (Fig. 1A,C). When a culture flask containing a subconfluent monolayer had been incubated on a RPM for 7 days, two types of Nthy-ori 3–1 cell populations could be distinguished. They grew either within a monolayer adhering to the bottom of the culture flaks (RPM AD), or within detached cells, which formed MCS (RPM MCS) and are floating in the supernatant (Fig. 1B). However, in the samples exposed for 14 days, no further increase in the number of MCS was detectable, whereas the size of the individual MCS had clearly increased (Fig. 1D).

#### FTC-133

All static 1*g*-control samples showed no spheroid formation (Fig. 1E,G). After a 7- and 14-day-exposure on the RPM, numerous MCS were formed in addition to a monolayer of adherent cells (Figure F, H). In the 14-day samples, an even enhanced number of MCS was clearly visible. In contrast to the Nthy-ori 3–1 cell line, which was not producing more MCS after 14 days, FTC-133 showed a steady increase of MCS (Fig. 1H).

### Simulated microgravity changes the cytoskeleton

To get a deeper understanding of the 3D spheroid formation, the actin filament (F-actin) system of the samples was investigated after a 7-day-exposure (Fig. 1I–N). Both cell lines showed comparable F-actin patterns in 1*g*-control adherent cells (Fig. 1I,L) as well as in RPM AD cells (Fig. 1J,M) and RPM MCS (Fig. 1K,N). In 1*g*-control cells, F-actin presented an equal distribution and an accumulation towards the cell membrane (Fig. 1I,L). In contrast, the RPM AD cells demonstrated a perinuclear clustering of F-actin (Fig. 1J,M), but RPM MCS showed a diffuse and unorganized pattern. A thicker layer at the cell membrane was detectable (Fig. 1K,N).

**Figure 2:**
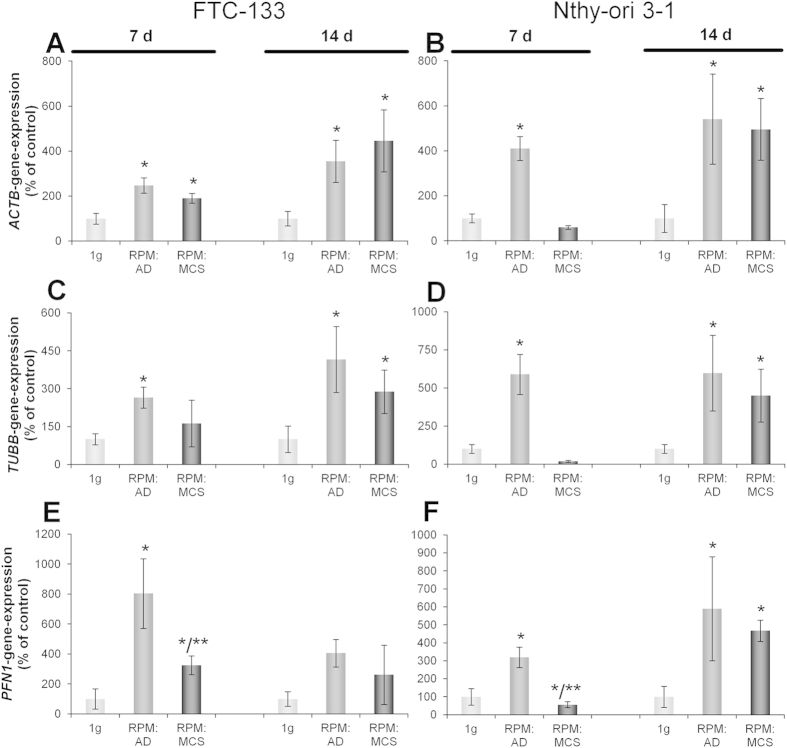
qPCR to determine the gene expression of cytoskeletal genes. *ACTB* (**A**,**B**): FTC-133 7d n = 4 and 14d n = 8, Nthy-ori 3-1 7d n = 5 and 14d n = 8. *TUBB* (**C**,**D**): FTC-133 7d n = 4 and 14d n = 8, Nthy-ori 3-1 7d n = 5 14d n = 8. *PFN1* (**E**,**F**) FTC-133 7d and 14d n = 4, Nthy-ori 3-1 7d n = 5 and 14d n = 4 *P < 0.05 vs. 1*g*, **P<0.05 AD vs. MCS.

In addition to the F-actin studies, the expression of *β-actin* gene (*ACTB*) was measured in static 1*g* cells, RPM AD cells and RPM MCS, respectively, after 7 and 14 days (Fig. 2A,B). After 7 days, FTC-133 RPM AD and RPM MCS cells showed a significant up-regulation of *ACTB* compared to the static 1*g*-control, whereas Nthy-ori 3–1 RPM-samples showed a significant up-regulation in RPM AD cells, but a down-regulation in RPM MCS. In contrast, both cell lines showed a significant *ACTB* mRNA up-regulation in RPM AD cells and RPM MCS after 14 days on the RPM.

Another important part of the cytoskeleton are the microtubules. Investigations on the gene expression of *β-tubulin* (*TUBB*) after 7 days of culturing on the RPM revealed a significant up-regulation in RPM AD cells of the FTC-133 and Nthy-ori 3–1cell line (Fig. 2C,D). In addition, the gene expression of *TUBB* was slightly elevated in FTC-133 cells, whereas Nthy-ori 3–1 cells showed a significant down-regulation of the *TUBB* mRNA. After 14 days, both cell lines showed an increased gene expression of *TUBB* in RPM AD cells as well as in spheroids (Fig. 2C,D).

Profilin is an important actin binding protein, which has an impact on actin polymerization. After a 7-day-exposure we found significantly elevated profilin mRNAs (*PFN1)* in FTC-133 RPM AD and RPM MCS cells (Fig. 2E). In contrast, Nthy-ori 3–1 showed a significant increase in RPM AD but a down-regulation in RPM MCS cells (Fig. 2F). After 14 days the gene expression in FTC-133 showed slight non-significant increases, however, the expression in Nthy-ori 3–1 was highly and significantly increased in RPM AD and RPM MCS compared to the 1*g*-control cells (Fig. 2F).

### Long-term simulated microgravity changes the mRNA expression of *VEGF,* its receptors and signalling pathway molecules in thyroid cells

In addition to the examination of the cytoskeleton, gene expression and release of signalling factors playing a role in angiogenesis and metastasis were investigated. VEGF is a secreted protein, which is important for angiogenesis and was examined on both transcriptional and secretory levels for changes during formation of 3D aggregates.

**Figure 3:**
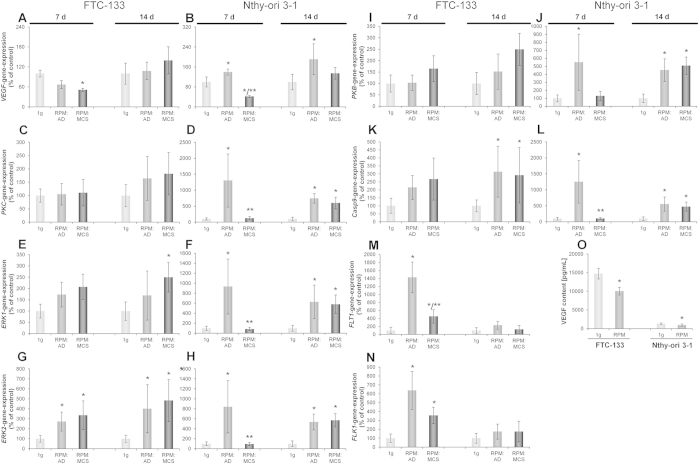
qPCR to determine VEGF, its receptors and signalling pathway molecules after 7 and 14 days on the RPM. Secretion of VEGF after 7 days. RPM: AD; adherent cells after RPM-exposure, RPM: MCS; multicellular spheroids formed after RPM-exposure. *VEGF* (**A**,**B**): FTC-133 7d n = 4 and 14d n = 8, Nthy-ori 3-1 7d n = 5 and 14d; n = 8. *PKC* (**C**,**D**): FTC-133 7d n = 4 and 14d n = 4, Nthy-ori 3-1 7d n = 4 and 14d n = 4. *ERK1* (**E**,**F**): FTC-133 7d n = 4 and 14d n = 4, Nthy-ori 3-1 7d n = 4 and 14d n = 4. *ERK2* (**G**,**H**): FTC-133 7d n = 4 and 14d n = 4, Nthy-ori 3-1 7d n = 4 and 14d n = 4. *PKB* (**I**,**J**): FTC-133 7d n = 4 and 14d n = 4, Nthy-ori 3-1 7d n = 4 and 14d n = 4. *Casp9* (**K**,**L**): FTC-133 7d n = 4 and 14d n = 4, Nthy-ori 3-1 7d n = 4 and 14d n = 4. *FLT1* (**M**): FTC-133 7d and 14d n = 4. *FLK1* (**N**): FTC-133 7d and 14d n = 4. VEGF protein released in the supernatant (**O**): FTC-133 n = 10, Nthy-ori 3-1 n = 10. LLD = 3 pg/ml. *P < 0.05 vs. 1*g* and **P < 0.05 vs. RPM: AD.

The *VEGF* gene expression in 7-day-samples revealed a mostly unchanged *VEGF* expression in FTC-133 RPM AD cells and a significant down-regulation in RPM MCS compared to the 1*g-*control (Fig. 3A). Nthy-ori 3–1 showed a significant up-regulation in RPM AD cells and a significant down-regulation in RPM MCS (Fig. 3B). After a 14-day-exposure FTC-133 cells exhibited a static *VEGF* expression in all groups (Fig. 3A), whereas Nthy-ori 3–1 cells showed a significant up-regulation in RPM AD cells and a *VEGF* mRNA level, comparable to 1*g*-controls (Fig. 3B).

After binding to vascular endothelial growth factor receptor 2 (VEGFR2; fetal liver kinase 1(FLK1)) several signalling cascades are activated which results in proliferation and survival of the cell. Upon phosphorylation of protein-kinase C (PKC), extracellular signal-regulating kinases (ERK) are activated which favour cell proliferation^27^. We found that after a 7- and 14-day-culture of FTC-133 on the RPM, the *PKC* gene-expression was not significantly changed (Fig. 3C). However, in Nthy-ori 3–1 cells a significant up-regulation of *PKC* in RPM AD cells after 7 days and significant up-regulations in RPM AD and RPM MCS cells after 14 days were found (Fig. 3D).

Examination of the gene expression of the downstream signalling molecules ERK1 and ERK2 revealed comparable results. After 7 and 14 days, FTC-133 showed an up-regulation of *ERK1* and *ERK2* in RPM AD and RPM MCS cells, even though only significant for *ERK1* after 14 days in RPM MCS. All RPM-cultures exhibited a significantly elevated *ERK2* compared with 1*g*-control samples (Fig. 3E,G). In contrast to the FTC-133 cell line, Nthy-ori 3–1 revealed a significant up-regulation of *ERK1* and *ERK2* in RPM AD cells but not in RPM MCS cells after 7 days. After 14 days, however, *ERK1* and *ERK2* were significantly up-regulated in both rotated cell populations (Figure F,H).

Another signalling pathway, which is activated by binding of VEGFR, inherits protein kinase B (PKB) acting on caspase-9 (Casp9) and favouring cell survival^27^. Investigating the *PKB* gene-expression of FTC-133 after 7 and 14 days showed an increased but not significant up-regulation (Fig. 3I). In contrast, Nthy-ori 3–1 cells exhibited a significant up-regulation of *PKB* in RPM AD after 7 days and in both cell populations after 14 days (Fig. 3J).

*Casp9* demonstrated a comparable gene-expression pattern as *PKB*. After 7 and 14 days, FTC-133 showed an elevated *Casp9* gene expression in RPM AD and RPM MCS samples, whilst being significant after 14 days (Fig. 3K). As for *PKB*, *Casp9* was only significantly up-regulated in RPM AD Nthy-ori 3–1 cells after 7 days, but significantly up-regulated in both cell populations after 14 days (Fig. 3L).

The corresponding receptors to VEGF are vascular endothelial growth factor receptor 1 (FMS-like tyrosine kinase 1 (FLT1)) and vascular endothelial growth factor receptor 2 (FLK1). Both were not detectable by quantitative real time polymerase chain reaction (qPCR) for the Nthy-ori 3–1 cell line. *FLT1* and *FLK1* (Fig. 3M,N) showed a significant up-regulation in RPM AD and RPM MCS in FTC-133 cells after 7 days. After 14 days however, there is a static expression of *FLT1* and *FLK1*.

Measuring the VEGF release into the medium after 7 days revealed a significantly lower content in RPM-samples of both cell types compared to the corresponding controls (Fig. 3O). Nthy-ori 3–1 1*g* cells only released a low amount of VEGF in the supernatants (1*g*: 1297 pg/mL vs. RPM: 910 pg/mL) compared with FTC cells (1*g*: 14740 pg/mL vs. RPM: 10051 pg/mL).

### Further factors promoting tumour growth and angiogenesis

Osteopontin is highly expressed in a variety of tumours^28^,^29^,^30^. It is known that osteopontin is a secreted, integrin-binding protein that contributes to tumour progression of several tumours^30^.

**Figure 4:**
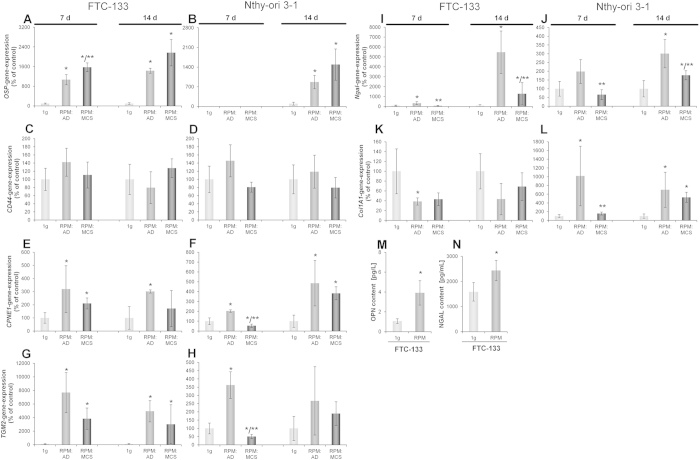
qPCR to determine the gene expression of factors promoting tumor growth after a 7- and 14-day-exposure on the RPM. Release of OPN and NGAL in the supernatant after a 7-day-exposure on the RPM. RPM: AD; adherent cells after RPM-exposure, RPM: MCS; multicellular spheroids formed after RPM-exposure. *OSP* (**A**,**B**): FTC-133 7d n = 4 and 14d n = 8, Nthy-ori 3-1 7d no quantification possible and 14d n = 8. *CD44* (**C**,**D**): FTC-133 7d n = 4 and 14d n = 4, Nthy-ori 3-1 7d n = 5 and 14d n = 4. *CPNE1* (**E**,**F**): FTC-133 7d n = 4 and 14d n = 4, Nthy-ori 3-1 7d n = 5 and 14d n = 4. *TGM2* (**G**,**H**): FTC-133 7d n = 4 and 14d n = 4, Nthy-ori 3-1 7d n = 5 and 14d n = 4. *NGAL* (**I**,**J**): FTC-133 7d n = 4 and 14d n = 4, Nthy-ori 3-1 7d n = 5 and 14d; n = 4. *Col1A1* (**K**,**L**): FTC-133 7d n = 4 and 14d n = 4, Nthy-ori 3-1 7d n = 5 and 14d; n = 4. OPN protein release in the supernatant (**M**): FTC-133 n = 10, NGAL protein secretion (**N**) FTC-133 n = 10. *P < 0.05 vs. 1*g* and **P < 0.05 vs. RPM: AD.

The osteopontin (*OSP*) gene expression in FTC-133 cells was up-regulated in all RPM-samples (Fig. 4A). Nthy-ori 3–1, in contrast, showed no detectable *OSP* mRNA after 7 days in all samples, but a significantly up-regulated mRNA after a 14 day-exposure to the RPM (Fig. 4B). The secretion behaviour mirrored the expression pattern as FTC-133 showed an elevated osteopontin (OPN) content in the supernatant after 7 days; whereas Nthy-ori 3–1 had not released a detectable amount of the protein (Fig. 4M).

The cell-surface glycoprotein cluster of differentiation (CD) 44 interacts with a variety of molecules, acting in cell adhesion and cell-cell interaction. It is a receptor for OSP^31^. *CD44* mRNA expression was static and comparable for both cell lines after a 7- and 14-day-exposure on the RPM (Fig. 4C,D).

The copine 1 gene (*CPNE1*) expression is significantly up-regulated in FTC-133 after 7 days in RPM AD and RPM MCS and after 14 days in RPM AD cells (Fig. 4E). Nthy-ori 3–1 showed slightly different results (Fig. 4F). After 7 days RPM AD cells showed a significant up-regulation, but a down-regulation in RPM MCS. After 14 days however, RPM AD and RPM MCS were significantly up-regulated.

Tissue transglutaminase (*TGM2*) mRNA expression is highly and significantly up-regulated in all RPM samples in FTC-133 compared to the control (Fig. 4G). Nthy-ori 3–1 showed a significant up-regulation in RPM AD and a significant down-regulation in RPM MCS after 7 days. After 14 days the *TGM2* expression was static in Nthy-ori 3–1 cells (Fig. 4H).

The neutrophil gelatinase-associated lipocalin (*NGAL*) gene expression in FTC-133 after a 7-day-culture on the RPM (Fig. 4I) showed comparable results to the expression of *NGAL* of Nthy-ori 3–1 (Fig. 4J). Both demonstrated an up-regulation in RPM AD cells, which was significant in FTC-133 cells. In both cases, RPM MCS expressed a significant lower *NGAL* mRNA than RPM AD samples. After 14 days FTC-133 (Fig. 4I) as well as Nthy-ori 3–1 (Fig. 4J) exerted a significant up-regulated *NGAL* mRNA in RPM AD and RPM MCS.

Measuring the NGAL content of 7-day-supernatants revealed a significantly increased amount in RPM samples for FTC-133 (Fig. 4N), whereas no NGAL was detectable for Nthy-ori 3–1 samples.

Collagen 1 (Col1A1) is important for 3D-tissue formation. It is a major protein of the extracellular matrix^13^. *Col1A1* gene-expression was down-regulated for FTC-133 after 7 and 14 days in both cell populations (Fig. 4K). Nthy-ori 3–1 instead, exhibited a significant up-regulation of *Col1A1* after 7 days in RPM AD cells and showed a significant up-regulation of the gene in both RPM AD cells and RPM MCS after 14 days (Fig. 4L).

Matrix metalloproteinase 3 (MMP-3) was released in the supernatant by both cell lines (Table 1). MMP-3 was constantly released in FTC-133 cells after 7 days under both conditions, whereas an increase was measured in Nthy-ori 3–1 cells cultured on the RPM.

### Simulated microgravity induced an altered gene expression and secretion of cytokines

Table 1 gives an overview about the soluble factors detected by Multianalyte Profiling (MAP) technology using MAP A and B for the supernatants of both cell lines after a 7-day-exposure to the RPM. Interferon-gamma (IFN-gamma), the interleukins (IL-2, IL-3, IL-4, IL-5, IL-10, IL-18), macrophage inflammatory protein 1 beta (MIP-1 beta), and the tumour necrosis factors (TNF-apha and TNF-beta) were not detectable by MAP profiling in both cell lines.

Several cytokines such as interleukin-6 (IL-6) and interleukin-8 (IL-8) are known to be involved in angiogenesis and metastasis in different types of cancer. IL-8 is important in endothelial cell structure maintenance and angiogenesis^32^.

**Figure 5:**
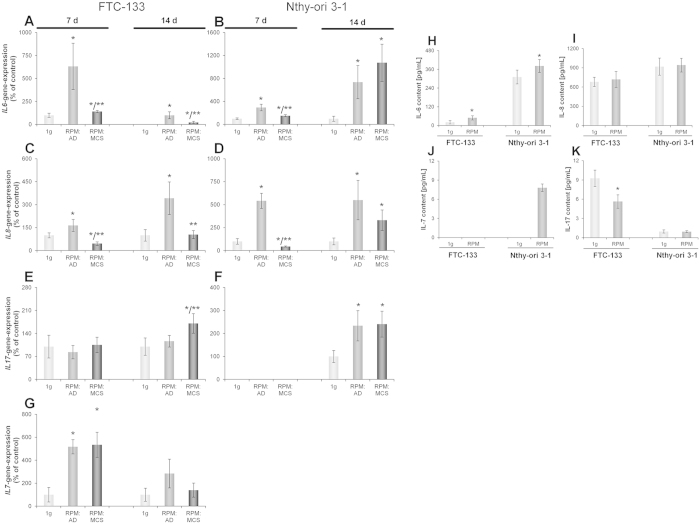
qPCR to determine the gene expression of cytokines after 7 and 14 days on the RPM. Release of cytokines after a 7-day-exposure on the RPM. RPM: AD; adherent cells after RPM-exposure, RPM: MCS; multicellular spheroids formed after RPM-exposure. *IL6* (**A**,**B**): FTC-133 7d n = 4 and 14d n = 8, Nth-ori 3-1 7d n = 5 and 14d n = 8. *IL8* (C,**D**): FTC-133 cells 7d n = 4 and 14d n = 8, Nthy-ori 3-1 7d n = 5 and 14d n = 8. *IL17* (**E**,**F**): FTC-133 cells 7d n = 4 and 14d n = 8, Nthy-ori 3-1 7d n = 5 and 14d n = 8. *IL7* (**G**): FTC-133 7d n = 4 and 14 days n = 8, for Nthy-ori 3-1 the signal was at the detection border, therefore no reliable quantification possible. IL-6 protein released in the supernatant (**H**): FTC-133 n = 10, Nthy-ori 3-1 n = 10. LLD = 1.0 pg/ml. IL-8 protein secretion (**I**): FTC-133 n = 10, Nthy-ori 3-1 n = 10. LLD = 0.56 pg/ml. IL-7 protein release (**J**): FTC-133 no release detectable, Nthy-ori 3-1 n = 10 except RPM n = 7. LLD = 6.1 pg/ml. IL-17 protein secretion (**K**): FTC-133 n = 10, Nthy-ori 3-1 n = 10. LLD = 0.31 pg/ml. *P < 0.05 vs. 1*g* and **P < 0.05 vs. RPM: AD.

Investigations of the *IL6* gene expression (Fig. 5A,B) of FTC-133 and Nthy-ori 3–1 cells after a 7- and 14-day RPM-exposure revealed a significant up-regulation of *IL6* in RPM AD cells compared with 1*g*-controls and RPM MCS. In fact, the *IL6* gene expression in RPM MCS was lower than in RPM AD cells, but still significantly higher than in the corresponding 1*g-*control. In addition, the secretion behaviour after 7 days (Fig. 5H) mirrored the gene expression pattern in FTC-133 as well as Nthy-ori 3–1 cells, which showed an elevated content of IL-6 in the cell culture medium.

The mRNA level of interleukin-7 (*IL7)* in FTC-133 cells was investigated after exposure to simulated microgravity for 7 and 14 days, respectively (Fig. 5G). *IL7* mRNA was up-regulated in RPM AD cells and RPM MCS after 7 days, but only elevated in RPM AD cells after 14 days. The *IL7* mRNA was not measurable in Nthy-ori 3–1 cells.

The gene expression of *IL8* (Fig. 5C,D) after a 7-day-exposure on the RPM revealed a significant up-regulation in RPM AD cells and a down-regulation in the RPM MCS for both cell lines, respectively. After a 14-day-culture on the RPM, FTC-133 and Nthy-ori 3–1 cells exhibited a comparable up-regulation of *IL8* in RPM AD cells. Interestingly, the *IL8* mRNA was only up-regulated in Nthy-ori 3–1 RPM MCS. In addition, the measurement of secreted IL-8 protein after 7 days revealed no changes in both cell types compared to the corresponding controls (Fig. 5I).

Interleukin-17 (*IL17*) mRNA was detectable after 7 days in FTC-133 cells. After 14 days the *IL17* mRNA level in RPM MCS was elevated compared with 1*g* and RPM AD samples (Fig. 5E). Nthy-ori 3–1, instead, showed no valid expression after 7 days, but an up-regulation after 14 days (Fig. 5F). The secretion of the cytokine was detectable in both cell types (Fig. 5K) with FTC-133 RPM samples showing a significantly lower content than the control samples.

The following soluble factors were measured in the supernatants of FTC-133 cells, but not in Nthy-ori 3–1 cells: Intercellular adhesion molecule 1(ICAM-1), interleukins (IL-1 alpha, IL-1 beta, IL-1ra, I-12p40, I-12p70, I-15, IL-23) and macrophage inflammatory protein 1 (MIP-1) alpha. Monocyte chemoattractant protein-1 (MCP-1) was constantly released by Nthy-ori 3–1 cells under both conditions (Table 1).

In addition, both cell lines released the following cytokines in the supernatant with different biological functions: Granulocyte-macrophage colony-stimulating factor (GM-CSF), brain-derived neurotrophic factor (BDNF), eotaxin-1, and stem cell factor (SCF). GM-CSF was significantly elevated in both cell types after RPM-exposure. Eotaxin-1 was not changed in all samples, and BDNF was elevated in Nthy-ori 3–1 RPM cultures. SCF was significantly elevated in FTC-133 RPM cultures and unchanged in Nthy-ori 3–1 RPM cultures (Table 1).

### Interactions of the investigated proteins

**Figure 6:**
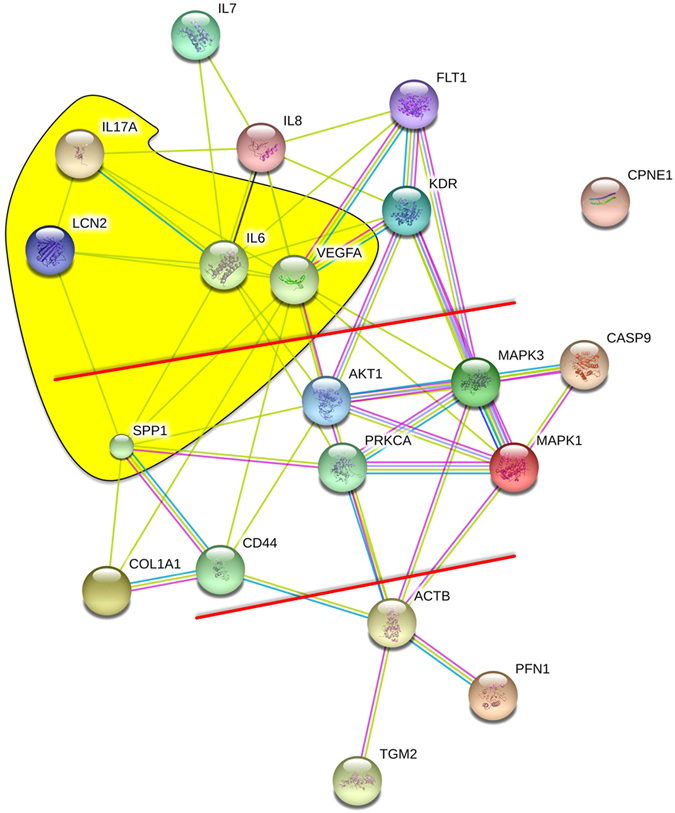
String analysis of proteins/genes analysed in this study. The network of interaction can be divided in three parts. Above the upper red line, a cluster of cytokines and some of their receptors can be seen. A cluster of cytoskeletal proteins is visible below the lower red line. Between the two red lines, kinases and membrane-binding proteins are shown, which may mediate the effects of the cytokines on the cytoskeleton. The entities shown on yellow background indicate proteins/genes, which are regulated in different ways, when malignant and normal thyroid cells form spheroids. Additional proteins: KDR: Kinase insert domain receptor, LCN2: Lipocalin 2, and SPP1: secreted phosphoprotein 1 (OSP).

After we had detected the microgravity-dependent changes of mRNA expression and factor secretion, we were interested to see whether these events of alteration are single isolated events or whether they are processes of regulation, which could influence each other. For this purpose, we entered the Swissprot numbers of the factors indicated in Table 2 in Search Tool for the Retrieval of Interacting Genes/Proteins (STRING) and focused on interactions. The results shown in Fig. 6 clearly visualize a network of interaction, which can be divided in three parts. A cluster of cytokines and some of their receptors can be seen in the upper part, and a cluster of cytoskeletal proteins is visible in the lower part of Fig. 6. Both clusters are connected via clusters of kinases and in parallel via clusters of membrane-binding proteins. Hence, it appears that kinases and membrane-binding proteins may mediate the effects of the cytokines on the cytoskeleton.

## Discussion

### Impact of simulated microgravity on cell growth

Devices designed to simulate microgravity conditions on Earth like the Rotating Wall Vessel or the RPM proved to be useful for tissue engineering^3^,^10^,^33^. Apart from simulated microgravity, several other methods to tissue-engineer spheroids such as the hanging drop technique, the carboxymethyl cellulose as well as spinnerflask method or the liquid overlay technique^34^,^35^ are known, but there is a lack of systematic studies evaluating the performance of these techniques. Moreover, these methods also do not reproduce the real tissue formation procedure. Real organ culture is not without its own problems; with difficulties in obtaining specimens and a poor viability of the tissues in culture being major obstacles. As animal models and *in vivo* studies are costly and complex with problems of unpredictable characteristics and ethical approval, physiological 3D model systems using human cells to create an authentic model is an obvious choice. With the recent advances in tissue engineering, 3D cultures are now more morphologically and functionally differentiated and can be produced in larger quantities as a result of greater control over culture composition and greater choice in the method of inducing 3D growth.

Here we show that the RPM promoted a scaffold-free formation of MCS of normal thyroid cells like it induced spheroid formation in poorly differentiated thyroid cancer cells and in chondrocytes^36^,^37^ earlier. However, we found a different growth behaviour for both cell lines. After a 7-day-exposure of FTC-133 on the RPM large and numerous MCS were found, which even increased in size during the following week. In contrast, the Nthy-ori 3–1 cells produced many small MCS in 7 days. They did not further grow in number until 14 days, but only in size. The different findings for spheroid growth could be explained by the nature of the cell lines (highly aggressive cancer cell line vs. benign thyroid cell line) and their inherent basic growth factor production and secretion (Fig. 3).

F-actin staining indicated cytoskeletal changes, which have already been described for various cell types, like chondrocytes, thyroid cancer cells, endothelial cells, osteoblasts, and others after different time periods of exposure to real and simulated microgravity^12^,^38^,^39^,^40^,^41^. The cytoskeletal structure of all these cell types reacted in a similar way to microgravity. This strongly suggests that the cytoskeleton may act as a general gravi-sensor and that the cytoskeleton remodelling process may play a key role for 3D growth of each cell type.

Beta-actin is one of six different actin isoforms and a non-muscle cytoskeletal actin. Because the actin proteins are involved in cell motility, structure and integrity, we have focused on the *ACTB* gene expression. In FTC-133 cells an increase in *ACTB* mRNA was detectable in both RPM AD and RPM MCS samples at both time periods compared with static 1*g*-controls. In Nthy-ori 3–1 cells a non-significant down-regulation of *ACTB* was found in RPM MCS samples after 7 days. The *ACTB* up-regulation may be due to gravitational loading and induce a shape deformation, which can result in 3D formation. The down-regulation of *ACTB* after 7 days on the RPM in Nthy-ori 3–1 MCS might also be a reason why the size of the spheroids is not as big as the size of FTC-133 spheroids. Alterations of the cellular architecture can trigger cellular responses to their environment^42^.

Similar results were obtained for the quantification of *TUBB* mRNA. A *TUBB* up-regulation was found in RPM AD and RPM MCS in 7- and 14-day FTC-133 samples compared with 1*g-*controls. *TUBB* gene expression was blunted in Nthy-ori 3–1 RPM MCS after a 7-day-exposure. Earlier it was shown that microtubules struggle to build up *in vitro* when exposed to a microgravity environment^42^. The results could be explained as follows: due to gravitational unloading, the shape of the AD cells is changed. To counteract the deformation, the cell reacts by an up-regulation of *β-tubulin*. The difference between RPM AD cells and RPM MCS could be due to the different stages. The AD cells fight to maintain their 2D-cell layer and to grow adherently while the cells in MCS are already growing three-dimensionally.

Profilin-1 is mainly responsible for actin fibre polymerization and reacts to extracellular signals^43^. An over-expression of *PFN1* suppresses micro-metastasis of MDA-MB-231 breast cancer cells in nude mice. In addition, an up-regulation of *PFN1* could inhibit mobility and invasion in breast cancer^44^. In contrast, profilin-1 was found to be over-expressed in renal cell carcinoma (RCC), indicating its potential as a diagnostic or progression biomarker and a possible target in RCC^45^. Long-term culture of FTC-133 cells on the RPM, resulted in an up-regulation of *PFN1* mRNA after 7 days in RPM AD cells and RPM MCS, whereas after 14 days there was a static expression. This might indicate a tumour-suppressive ability of microgravity, which was also described in different experiments^6^,^46^,^47^.

### Factors involved in tumour growth and angiogenesis

VEGF is a very important factor for 3D aggregation and is involved in angiogenesis and tumourigenesis and is of special interest because it is mitogenic, angiogenic, and a potent mediator of vascular permeability as well as essential to grow tissue^48^,^49^,^50^,^51^. The amount of secreted VEGF protein in FTC-133 cells was about 12-fold higher than in Nthy-ori 3–1 cells. This may be the major reason for the different morphological appearance shown in Fig. 1. However, the involvement of other growth factors, such as Epidermal Growth Factor and Connective Tissue Growth Factor^18^,^52^ has to be considered. In both types of cells the VEGF in sample supernatants was lower, when they were exposed to simulated microgravity for 7 days compared with their relevant 1*g*-controls. An extremely low VEGF in RPM samples may induce the enhancement of *VEGF* gene expression in RPM AD cells of Nthy-ori 3–1 and might be one reason why this cell line is able to produce MCS on the RPM at all. Previous experiments, investigating VEGF in FTC-133 for short-term (31 parabolas) and long-term (10 days) studies in real microgravity revealed an up-regulation of *VEGF* after 31 parabolas and a down-regulation after 10 days of real microgravity in Space^17^ for FTC-133 cells. These results are in agreement with the RPM findings and it may be concluded that VEGF is in part responsible for the early development of MCS, but does not participate in later stages of 3D growth.

FLT-1 (VEGFR-1) and FLK-1 (VEGFR-2) are the major receptors of VEGF^53^ and are expressed on endothelial cells as well as on a variety of tumours^54^. VEGFR-2 is suggested to be the major mediator of endothelial cell mitogenesis, survival, angiogenesis and vascular permeability. In contrast, VEGFR-1 seems not to be involved in mediating mitogenic effects on endothelial cells rather than blocking the mitogenic effect by sequestering with VEGF^55^. After a 7- and 14-day-exposure only FTC-133 demonstrated *VEGFR* expression. After 7 days *VEGFR-1* and *VEGFR-2* were significantly up-regulated in RPM AD and RPM MCS. After 14 days however, the expression was static. These results are in concert with findings, that VEGFR-1 is especially needed in early development^55^.

Upon binding of VEGF to its corresponding receptors, several signalling pathways are activated (Fig. 6). Phosphorylation of PKC is followed by activation of ERK1 and ERK2, which in the end favours cell proliferation^27^. We found *PKC* gene-expression to be only slightly up-regulated in FTC-133 cells after 14 days. In contrast, Nthy-ori 3–1 cells rotated for 7 days showed a highly up-regulated *PKC* gene expression in adherent cells (AD) and a significant up-regulation in both cell populations after 14 days. These findings are in concert with the results obtained for the gene-expression of the downstream molecules ERK1 and ERK2 demonstrating comparable results for both cell lines. Thus, it can be concluded that culturing cells on the RPM favours the proliferation of cells. The differences between the cell lines might also explain why the MCS of Nthy-ori 3–1 are much smaller than the ones of FTC-133.

Another signalling pathway which is activated by VEGF binding inherits PKB which negatively regulates caspase-9, resulting in cell survival. PKB-Caspase-9 demonstrated a comparable picture as PKC-ERK1/ERK2. Thus, the up-regulation of *Casp9* hints to an increased apoptotic behaviour of the cells. The apoptotic effect of microgravity was already demonstrated using the follicular thyroid carcinoma cell line ML-1 for 24 h and 48 h^6^.

In this study, we additionally investigated several proteins, which are known to be involved in cellular growth and differentiation. We focused on *CD44*, which is a multifaceted transmembrane glycoprotein. It establishes transmembrane complexes and organizes signalling cascades via association with the actin cytoskeleton. It is able to monitor changes in the extracellular matrix and triggers cell growth, survival and differentiation^56^. Interestingly, after RPM-exposure, the expression of *CD44* was unaffected. Nevertheless, a role of CD44 in the perception of gravity should be considered. It can be activated by OPN, whose presence is increased (Fig. 4A,B,M), to modify integrin interaction with the extracellular matrix^57^. Moreover, the elevated IL-6 concentrations (Fig. 5A,B,H) could be due to activated CD44 receptors (Fig. 6)^58^. This suggests that *CD44* might be involved in the perception of altered gravity, having an actin cytoskeleton remodelling effect, although its mRNA concentration is not changed.

In the next step, we focused on copine 1. Copine 1 is expressed in a variety of different tissues, such as brain, heart, lung, liver and kidney^59^. It was shown that copine 1 plays a pivotal role in regulating cell growth, apoptosis, and metastatic functions. An over-expression of copine 1 was linked to abolishing nuclear factor 'kappa-light-chain-enhancer' of activated B-cells (NF-κB) transcription^60^. *CPNE1* mRNAs are clearly up-regulated in both cell lines after RPM-culture so that we will focus on NF- κB signalling in future studies.

Another factor playing a role in 3D growth and metastasis is TGM2, which is highly over-expressed in a variety of tumours^61^. *TGM2* is a stress-responsive gene and its expression is frequently up-regulated during inflammation and wounding^61^. A forced or basal high expression of *TGM2* leads to a constitutive activation of NF- κB^62^. *TGM2* expression is associated with poor disease outcome, increased drug resistance, and increased incidence of metastasis. In FTC-133 *TGM2* is highly up-regulated upon RPM-exposure after 7 and still after 14 days. In contrast, Nthy-ori 3–1 cells only showed an over-expression after 7 days and a static expression after 14 days. These results suggest that *TGM2* has an impact on the formation and proliferation of MCS in general, irrespective of malignant or non-malignant cells. The static expression in Nthy-ori 3–1 cells after 14 days might also be the reason for smaller diameters of MCS and lower MCS numbers.

Measurement of the IL-6 content after the experimental procedure revealed a much higher level in the supernatant of the Nthy-ori cells and a significant increase in all RPM-samples of both cell lines compared with the corresponding 1*g-*controls. The cytokine IL-6 is an important factor in angiogenesis and metastasis. IL-6 has the capability to act on every cell type and is known to be highly elevated in some tumour types^63^. As IL-6 is a potent factor triggering survival and proliferation via osteopontin, PKC, PKB and VEGF (Fig. 6). It is of special interest concerning the development of 3D-aggregates^3^. An increase in *IL6* mRNA was found in RPM adherent (AD) cells of both cell lines at both time points. In MCS *IL6* was down-regulated in both cell lines after a 7-day-exposure. In contrast to the results in this study, experiments using human endothelial cells displayed no changes concerning IL-6 although 3D-aggregates were formed, but these 3D structures were a mixture of intima constructs (tubes) and MCS^64^. Taken together, it can be suggested that the IL-6 secretion is cell-type-dependent, but has a strong impact on continuous growth of adherent cells under microgravity. It has been shown in phase II studies, that the extent of increase in IL-6 in the plasma during treatment was associated with an inferior outcome in patients with rectal and ovarian cancer after bevacizumab and chemoradiation treatment^65^, and with an inferior outcome in patients with advanced hepatocellular carcinoma after sunitinib therapy^66^.

IL-8 has an influence on proliferation, survival, angiogenesis and invasion^67^. For this reason, IL-8 might also play a role in the development of 3D-aggregates. The *IL8* gene expression in both cell lines and at both time points was similar. There was an increase of *IL8* in RPM AD cells and a down-regulation in MCS. The release of IL-8 protein was similar in FTC-133 and Nthy-ori 3–1 samples and did not change in RPM samples compared with 1*g-*samples of both cell lines at day 7. In contrast to data in this study, experiments with FTC-133 cells on the RPM and in Space for 10 days presented a down-regulation of *IL8* in AD cells and MCS as well as a significantly lower IL-8-content in the supernatant^17^. It can be concluded that the IL-8 expression and secretion occurs in a time-dependent manner.

The discovery of IL-17 was a coincidence, as the multianalyte profiling (MAP) included the test for this cytokine. IL-17 is secreted by specialized Th17 cells of the immune system^68^. That is why it is surprising that *IL17* mRNA is expressed by thyroid cells. Why thyroid cells express *IL17* is not known to date, however, simulated microgravity using the RPM has an impact on the expression of this cytokine and might contribute to the expression of IL-6 and IL-8^69^. The proinflammatory cytokine IL-17 plays a potential role in T-cell-mediated angiogenesis and promotes tumourigenicity of human cervical cancer. The expression of IL-17 in human colorectal cancer was reported in 2006^70^. It has not yet been reported that IL-17 is secreted by thyroid cells. Both cell lines released the IL-17 protein in the supernatant after 7 days.

We focused on the cell adhesion molecule osteopontin, which is known to play an essential role in two key aspects of tumour progression: VEGF expression by tumour cells and VEGF-stimulated neovascularization^71^. Osteopontin is highly expressed in a variety of tumours^28^.

Follicular thyroid carcinoma cells are constantly producing osteopontin, but conditions of simulated microgravity might enhance the production of this protein. After 7 and 14 days we observed a clear increase of *OSP* mRNA in RPM AD cells and RPM MCS compared with 1*g-*samples in FTC-133 cells. This might explain the huge FTC-133 tumour spheroids. *OSP* mRNA was detectable after 14 days in Nthy-ori 3–1 cells and clearly elevated in RPM MCS. Nthy-ori 3–1 cells seem to react to simulated microgravity much later with a production of osteopontin, which might also influence the size of the spheroids.

A 24-hour-exposure of FTC-133 cells to conditions of simulated microgravity resulted in an up-regulation of *OSP* in RPM AD cells but in a down-regulation in RPM MCS^10^. Ten days of real microgravity induced a down-regulation of *OSP* in AD cells and MCS, but using an RPM for 10 days^17^ mirrors the results obtained in this work. A reason for this may be cosmic radiation in Space, as well as vibration or hypergravity effects during launch have to be taken into account. Based on its role as mediator of cell-matrix adhesion and communication, osteopontin has the potential to profoundly influence tumourigenesis and invasion^72^ and thus, also 3D growth.

NGAL is a glycoprotein and seems to represent a marker of malignant follicular cell-derived thyroid tumours^73^. In malignant cells, NGAL seems to inhibit apoptosis (in thyroid cancer cells), invasion and angiogenesis (in pancreatic cancer) as well as to enhance proliferation and metastasis (in breast and colon cancer)^74^. The NGAL protein release was significantly increased in FTC-133 cells compared with 1g-control cells, whereas Nthy-ori 3–1 cells did not secrete the protein at day 7. In SKOV-3 ovarian cancer cells, the levels of VEGF and IL-6 were decreased after NGAL silencing, suggesting that NGAL controls VEGF and cytokine production and it may be common among cancer cell types^75^. This should be investigated in future experiments to clarify whether this is a novel mechanism through which NGAL may promote tumour angiogenesis and progression.

The extracellular matrix protein collagen-1 was postulated to be important for the formation of 3D-aggregates whilst being enhanced after 1 up to 5 days of simulated microgravity^6^,^76^. We found *Col1A1* to be down-regulated for FTC-133 cells after 7 and 14 days, but up-regulated in the non-malignant cells. In an earlier study, investigating the ML-1 cell line (another poorly differentiated follicular thyroid cancer cell line) for 24 and 48 h on the 3D clinostat we measured an increase in the production of several extracellular matrix proteins (collagen I and III, laminin, fibronectin, chondroitin sulfate) compared with controls^6^. The Proteomics results of the recent spaceflight experiment Cellbox-1 revealed in a subsequent protein analysis 180 proteins by mass spectrometry^77^. These proteins suggested that the increased protein synthesis related to the ECM could detain the cells from MCS formation, when the cells are completely confluent at the time point, when the microgravity experiment starts^77^. Short-term microgravity during a parabolic flight altered the gene expression of extracellular matrix proteins and cell adhesion molecules: *COL*4A5 mRNA was down-regulated under microgravity, whereas *OPN* and *FN* were significantly up-regulated^11^. It was shown that the gravi-response of ML-1 cells occurred very early, within the first few seconds. These findings suggest a time-dependent and cell type-dependent regulation, which might also explain the smaller MCS formed by the non-malignant cell line.

### Additional soluble factors released in the supernatant

As detected by MAP profiling several cytokines were released by FTC-133 in the supernatant after 7 days (Table 1). An increased secretion was measured for GM-CSF, MIP-1 alpha, interleukins (IL-1 beta, IL-1ra, IL-12p40, IL-15) and SCF. In contrast, the release of MMP-3, ICAM-1, BDNF, Eotaxin-1, IL-1alpha, Il-12p70, and IL-23 in FTC-133 cells remained unchanged. Nthy-ori 3–1 cells released a similar amount of MCP-1 protein under both gravity conditions. These results will be further analysed in future studies and compared with future spaceflight data.

## Conclusions

Taken these results together, we could show that both cell types form 3D aggregates on the RPM after long-term exposure. But speed and extensions are different. There is an influence between the cytoskeleton and the cytokines mediated by kinases and/or membrane binding proteins, as well as a regulation of cytokines among themselves (Fig. 6). This study also suggests that growth and angiogenic factors determine differences in spheroid formation behaviour of the malignant and the healthy thyroid cells. Cytoskeleton dynamics are regulated by IL-8^67^ as well as by osteopontin^28^. The differential expression of these cytokines may contribute to the morphological appearance of the cells exposed to simulated microgravity on the RPM. In addition, IL-17 has a regulatory impact on IL-8 and IL-6 expression, which in turn regulates the expression of VEGF (Fig. 6). Moreover, NGAL may influence and control VEGF and cytokine production in thyroid cancer cells grown under conditions of microgravity. Taking into account that the non-malignant cell line does not express the VEGFRs in a measurable amount at the investigated time points, however, reacts with an up-regulation of downstream signalling molecules, may hint either to a delayed reaction to the simulated microgravity or to further signalling molecules which are involved in 3D-aggregate formation. How NGAL and VEGF interact in detail to transform adherent cells to 3D-aggregates has to be investigated in future studies. IL-6 could be a candidate that is influencing the cytoskeleton via ACTB (Fig. 6).

In summary, it can be concluded that the formation of multicellular spheroids is occurring in thyroid cancer cells as well as in normal cells, a finding, which shows that simulated microgravity is a usable approach for engineering functional tissue. These functional tissues might be used in the future for pharmacological testing or co-culture experiments to investigate neoangiogenesis inhibitors, yielding a more accurate picture than mouse or rat models.

## Methods

### Cell culture

Nthy-ori 3–1 primary human thyroid follicular epithelial cells and FTC-133 human follicular thyroid carcinoma cells were grown in RPMI 1640 (Life Technologies, Naerum, Denmark) medium supplemented with 10% fetal calf serum (Biochrom AG, Berlin, Germany) and 1% penicillin/streptomycin (Life Technologies) and cultured under standard cell culture conditions at 37 °C and 5% CO_2_. One day prior to the RPM experiment, 1 × 10^6^ cells were counted and seeded into T25cm^2^ vented cell culture flasks (Sarstedt, Newton, USA) or 2.5 × 10^5^ cells were seeded into slide flasks (Thermo Scientific, Roskilde, Denmark) for cytoskeleton investigations. To avoid shear stress, each flask was completely filled with medium, taking care that no air bubbles remained in the cell culture flasks. The flasks were installed on the RPM and run for the time and mode of interest. Corresponding static 1*g-*controls were prepared in parallel and were stored next to the device in the same incubator. During the 14-day-experiments, the medium had to be changed after 7 days to ensure the exchange of waste and nutrients. The flasks were positioned upright for 15 minutes to allow sedimentation of MCS. Afterwards, the old medium was aspirated carefully to ensure no disturbance and refilled with fresh medium. Then the flasks were installed on the devices to continue the experiment. At day 7 and day 14, the cells were investigated and photographed. The supernatant was collected and centrifuged at 4 °C to collect the MCS. After centrifugation the supernatant was collected for cytokine investigation on ice and then frozen at −20 °C. The MCS were collected and stored in liquid nitrogen.

To harvest the adherent cells, 5 ml of ice-cold phosphate buffered saline (PBS, Life Technologies) were carefully added to each T25 cm^2^ flask and the cells were scraped off with a scraper. The cell suspension was collected and centrifuged at 4 °C. The PBS was discarded and the dry pellet was stored in liquid nitrogen.

### Random Positioning Machine (RPM)

The RPM (Airbus Defense and Space, former Dutch Space, Leiden, Netherlands) was located at 37 °C and 5% CO_2_ in a commercially available incubator. The method was intensively investigated and published earlier^21^,^78^. The RPM was used in real random mode with random speed and random interval and a maximum speed of 75 °/s. Fifteen T25 cm^2^ flasks were fixed to the central frame, resulting in a maximum distance of 7.5 cm to the rotation axis, and were rotated for the time periods of interest (7d and 14d). 1*g* static controls were exposed to the same environmental conditions nearby the device, however, without rotation.

### Phase contrast microscopy

Phase contrast microscopy was performed for visual observation of the morphology of the cells, using a Leica (Microsystems GmbH, Wetzlar, Germany). Pictures had been taken by a Canon EOS 550D camera (Canon GmbH, Krefeld, Germany).

### F-Actin cytoskeleton staining

After a 7-day-exposure to the RPM, the medium was discarded and the slides were washed three times with 1× PBS. Then, the cells were fixed with an appropriate volume of 4% paraformaldehyde in PBS for 30 minutes at room temperature. The fixative was discarded and the cells were washed three times with 1× PBS for 5 minutes. Afterwards, blocking took place, using 1% bovine serum albumin (BSA) in PBS for 1 hour. After additional washing with 1× PBS for 3 times, the cells were covered with Phalloidin Texas Red (Life Technologies) diluted in PBS supplied with 0.1% BSA overnight. The next day, the cells were washed three times with PBS and covered with 4′,6-diamidino-2-phenylindole (DAPI; Life Technologies) diluted in PBS for 10 minutes. Before mounting, the cells were washed twice with PBS and once with distilled water. They were then mounted with glycerol and covered with a cover slip. The method was published in^12^,^13^.

### Cytokine measurements by Multi-Analyte Profiling technology

The collected supernatant was sent to Myriad RBM, Austin, Texas, USA. In order to detect cytokines of interest, two sets of analyses were done: Human CytokineMAP® A, and Human CytokineMAP® B. In short, a Multi-Analyte Profiling is performed which uses micro beads. These beads carry specific antibodies, which are directed against the target of interest. The secondary antibody is coupled to a biotinylated reporter. Binding is followed by an excess exposure to streptavidin phycoerythrin solution to develop the multiplexes. This enables the quantification of the target of interest via fluorescence detection^52^,^79^.

### OPN, NGAL, and VEGF measurements

OPN levels were determined using a commercial ELISA kit according to the manufacturer’s instructions (RayBiotech, Inc., GA, USA). The absorbance was measured at 450 nm. NGAL and VEGF levels were measured using in-house time resolved immunofluorometric assays (TRIFMA) according to previously described methods^79^,^80^. For all determinations, supernatant samples were diluted 1:2 and the 96-well plates were read using a VICTOR 2030 (Perkin Elmer, Inc.) Standard curves were used to calculate the concentrations using the standard software implemented in the VICTOR 2030.

### RNA isolation and quantitative real-time PCR

RNA isolation and quantitative real-time PCR were performed according to routine protocols^10^,^17^,^32^,^39^. RNA was isolated using the AllPrep RNA/Protein kit (Qiagen GmbH, Hilden, Germany) following manufacturer instructions. The RNA was quantified via Spectrophotometer Ultrospec 2100 pro (Amersham Biosciences, Amersham, Great Britain). Reverse transcription was performed using the First Strand cDNA Synthesis Kit (Thermo Scientific, Waltham, Massachusetts, USA) following manufacturer’s instructions. Quantitative real-time PCR was utilized to determine the expression levels of target genes, shown in Table 2, using the SYBR® Select Master Mix (Applied Biosystems, Darmstadt, Germany) and the 7500 Real-Time PCR System (Applied Biosystems). cDNA-selective Primers were designed to span exon-exon boundaries and to have a Tm of 60 °C using Primer Express software (Applied Biosystems), and were synthesized by TIB Molbiol (Berlin, Germany). All samples were measured in triplicate and normalized to the housekeeper 18S rRNA. Comparative threshold cycle (ΔΔC_T_) methods were used for relative quantification of transcription levels, with 1*g* set as 100%.

### STRING analysis

Interactions between the various factors were determined using the STRING platform^81^. For each protein, the UniProtKB entry number was inserted in the input form “multiple proteins” and “Homo sapiens” was selected as organism. The resulting network view was downloaded in the confidence view showing lines between interacting proteins^82^.

### Statistical Evaluation

Statistical Evaluation was performed using SPSS 15.0 (SPSS, Inc., Chicago, IL, USA). The Mann-Whitney-U-Test was used to compare 1*g* and s-μ*g* conditions, as well as AD cells and MCS cells. All data is presented as mean ± standard deviation (SD) with a significance level of p < 0.05. *indicating the comparison of 1*g* vs. AD, MCS and **representing the comparison of AD vs. MCS.

## Additional Information

**How to cite this article**: Kopp, S. *et al.* Mechanisms of three-dimensional growth of thyroid cells during long-term simulated microgravity. *Sci. Rep.* **5**, 16691; doi: 10.1038/srep16691 (2015).

## Figures and Tables

**Table 1:** Soluble factors detected by MAP A and B analyses after cultivation of FTC-133 and Nthy-ori 3-1 on the RPM for 7 days.

Factors MAP A	LDD (pg/ml)	FTC-133		Nthy-ori 3-1	
		7 days 1 *g* (pg/ml)	7 days RPM (pg/ml)	7 days 1 *g*(pg/ml)	7 days RPM (pg/ml)
GM-CSF	3.5	66.7 ± 8.3	264.6 ± 34.1*	11.52 ± 1.21	14.3 ± 1.7*
IFN-gamma	0.30	n.d.	n.d.	n.d.	n.d.
IL-2	5.7	n.d.	n.d.	n.d.	n.d.
IL-3	0.0010	n.d.	n.d.	n.d.	n.d.
IL-4	8.4	n.d.	n.d.	n.d.	n.d.
IL-5	0.70	n.d.	n.d.	n.d.	n.d.
IL-6	1.0	20.67 ± 11.31	49.2 ± 14.24*	311.4 ± 1.19	382.3 ± 44.07*
IL-7	6.1	n.d.	n.d.	8.26 ± 1.91	7.81 ± 0.64
IL-8	0.56	681.2 ± 75.9	719.6 ± 130.1	918.5 ± 140.3	942.4 ± 112.4
IL-10	0.66	n.d.	n.d.	n.d.	n.d.
IL-18	4.7	n.d.	n.d.	n.d.	n.d.
MIP-1 alpha	2.4	10.45 ± 1.55	14.29 ± 3.58*	n.d.	n.d.
MIP-1 beta	3.5	n.d.	n.d.	n.d.	n.d.
MCP-1	15	n.d.	n.d.	1144.6 ± 283.7	1255 ± 438.3
TNF-alpha	5.2	n.d.	n.d.	n.d.	n.d.
TNF-beta	6.4	n.d.	n.d.	n.d.	n.d.
Factors MAP B					
BDNF	0.0054	0.034 ± 0,0044	0.04 ± 0.0091	0.188 ± 0,033	0.237 ± 0.0134*
Eotaxin-1	13	74.6 ± 0.11	69.60 ± 10.37	17.80 ± 1.64	17.0 ± 2.78
ICAM-1	0.65	1.76 ± 0.11	2.04 ± 0.38	n.d.	n.d.
IL-1 alpha	0.00030	0.001 ± 0.0001	0.0013 ± 0.00036	n.d.	n.d.
IL-1 beta	0.44	1.18 ± 0.15	1.5 ± 0.31*	n.d.	n.d.
IL-1ra	31	252.20 ± 29.32	344.4 ± 97.89*	n.d.	n.d.
IL-12p40	0.019	0.17 ± 0.01	0.23 ± 0.05*	n.d.	n.d.
IL-12p70	6.8	26 ± 3.09	24.40 ± 6.67	n.d.	n.d.
IL-15	0.039	0.21 ± 0,02	0.26 ± 0,03*	n.d.	n.d.
IL-17	0.31	9.26 ± 1.34	5.64 ± 1.11*	0.97 ± 0.26	0.95 ± 0.17
IL-23	0.13	0.96 ± 0.12	1.16 ± 0.34	n.d.	n.d.
MMP-3	0.0052	0.102 ± 0.028	0.13 ± 0.024	0.022 ± 0.004	0.054 ± 0.006*
SCF	13	86.90 ± 9.52	126.40 ± 34.68*	27 ± 4.24	29 ± 6.67
VEGF	3.9	14740 ± 1489	10051 ± 1077*	1297.2 ± 231.2	938.9 ± 315.8*

Values are given with mean ± SD; 1*g*, corresponding static control; n.d., not detectable; n = 5; *P < 0.05 for device sample vs. corresponding 1*g* ground control; LDD (Least Detectable Dose)-determined as the mean ± 3 standard deviations of 20 blank readings.

**Table 2:** Primers used for quantitative real-time PCR.

Gene	Primer Name	Sequence
*18S rRNA*	18S-F	GGAGCCTGCGGCTTAATTT
	18S-R	CAACTAAGAACGGCCATGCA
*ACTB*	ACTB-F	TGCCGACAGGATGCAGAAG
	ACTB-R	GCCGATCCACACGGAGTACT
*Casp9*	Casp9-F	CTCCAACATCGACTGTGAGAAGTT
	Casp9-R	GCGCCAGCTCCAGCAA
*CD44*	hCD44-F	ACCCTCCCCTCATTCACCAT
	hCD44-R	GTTGTACTACTAGGAGTTGCCTGGATT
*COL1A1*	Col1A-F	ACGAAGACATCCCACCAATCAC
	Col1A-R	CGTTGTCGCAGACGCAGAT
*CPNE1*	CPNE1-F	CAGAGCTGAGGGATGATGACTTC
	CPNE1-R	TTTCCAGGCTTCAGCATCAA
*ERK1*	ERK1-F	ACCTGCGACCTTAAGATTTGTGA
	ERK1-R	AGCCACATACTCCGTCAGGAA
*ERK2*	ERK2-F	TTCCAACCTGCTGCTCAACA
	ERK2-R	TCTGTCAGGAACCCTGTGTGAT
*FLK1*	hFLK1-F	TCTTCTGGCTACTTCTTGTCATCATC
	hFLK1-R	GATGGACAAGTAGCCTGTCTTCAGT
*FLT1*	FLT1-F	CCCTCGCCGGAAGTTGTAT
	FLT1-R	GATAATTAACGAGTAGCCACGAGTCAA
*NGAL*	hNGAL-F	AGGGAGTACTTCAAGATCACCCTCTA
	hNGAL-R	AGAGATTTGGAGAAGCGGATGA
*IL6*	IL6-F	CGGGAACGAAAGAGAAGCTCTA
	IL6-R	GAGCAGCCCCAGGGAGAA
*IL7*	IL7-F	CCAGTTGCGGTCATCATGACTA
	IL7-R	TGATGCTACTGGCAACAGAACA
*IL8*	IL8-F	TGGCAGCCTTCCTGATTTCT
	IL8-R	GGGTGGAAAGGTTTGGAGTATG
*IL17*	IL15-F	CATCCAGTGCTACTTGTGTTTACTTCT
	IL15-R	CCAGTTGGCTTCTGTTTTAGGAA
*OPN*	OPN-F	CGAGGTGATAGTGTGGTTTATGGA
	OPN-R	CGTCTGTAGCATCAGGGTACTG
*PFN1*	hPFN1-F	GGGAATTTAGCATGGATCTTCGT
	hPFN1-R	ACCGTGGACACCTTCTTTGC
*PKB*	AKT1-F	CTTCTATGGCGCTGAGATTGTG
	AKT1-R	CAGCATGAGGTTCTCCAGCTT
*PKC*	PKC-F	CATTCAACAGCTGGGCAAGTT
	PKC-R	GTAGATGATGCCCTGATTGTGAAG
*TGM2*	hTGM2-F	AAGAGGAGCGGCAGGAGTATG
	hTGM2-R	GCCCAAAATTCCAAGGTATGTTC
*TUBB*	TUBB-F	CTGGACCGCATCTCTGTGTACTAC
	TUBB-R	GACCTGAGCGAACAGAGTCCAT
*VEGFA*	VEGFA-F	GCGCTGATAGACATCCATGAAC
	VEGFA-R	CTACCTCCACCATGCCAAGTG

All sequences are given in 5′-3′ direction.
